# Viscoelastic Behavior of Glass-Fiber-Reinforced Silicone Composites Exposed to Cyclic Loading

**DOI:** 10.3390/polym12091862

**Published:** 2020-08-19

**Authors:** Julia Beter, Bernd Schrittesser, Bernhard Lechner, Mohammad Reza Mansouri, Claudia Marano, Peter Filipp Fuchs, Gerald Pinter

**Affiliations:** 1 Polymer Competence Center Leoben GmbH, Roseggerstrasse 12, 8700 Leoben, Austria; Bernd.Schrittesser@pccl.at (B.S.); Bernhard.Lechner@pccl.at (B.L.); Mohammad.Mansouri@pccl.at (M.R.M.); PeterFilipp.Fuchs@pccl.at (P.F.F.); 2 Department of Chemistry, Materials and Chemical Engineering “Giulio Natta”, Politecnico di Milano, Piazza Leonardo da Vinci 32, 20133 Milan, Italy; claudia.marano@polimi.it; 3 Department of Polymer Engineering and Science, Montanuniversitaet Leoben, Otto Gloeckelstrasse 2, 8700 Leoben, Austria; Gerald.Pinter@unileoben.ac.at

**Keywords:** flexible composite, dynamic mechanical analysis, cyclic loading, step cycle test, viscoelasticity, fiber-reinforced elastomer

## Abstract

The aim of this work was to analyze the influence of fibers on the mechanical behavior of fiber-reinforced elastomers under cyclic loading. Thus, the focus was on the characterization of structure–property interactions, in particular the dynamic mechanical and viscoelastic behavior. Endless twill-woven glass fibers were chosen as the reinforcement, along with silicone as the matrix material. For the characterization of the flexible composites, a novel testing device was developed. Apart from the conventional dynamic mechanical analysis, in which the effect of the fiber orientation was also considered, modified step cycle tests were conducted under tensile loading. The material viscoelastic behavior was studied, evaluating both the stress relaxation response and the capability of the material to dissipate energy under straining. The effects of the displacement rate of the strain level, the amplitude of the strain applied in the loading–unloading step cycle test, and the number of the applied cycles were evaluated. The results revealed that an optimized fiber orientation leads to 30-fold enhanced stiffness, along with 10 times higher bearable stress. The findings demonstrated that tailored reinforced elastomers with endless fibers have a strong influence on the mechanical performance, affecting the structural properties significantly.

## 1. Introduction

Fiber-reinforced composites offer a synergetic combination of properties consisting of two or more individual components. Due to their beneficial interactions, completely new mechanical behavior can be generated, which cannot be achieved using the individual components [1]. This approach has been already applied very successfully to elastomers, where a specific improvement of mechanical properties is achieved via the use of fibrous reinforcement while still maintaining the high flexibility of the elastomeric matrix [2]. This enables higher bearable loadings while the good damping and absorption behavior are retained. In the industry, typical fiber-reinforced rubber products are used for several applications, such as automotive tires [3], dampers [4,5], conveyor belts [6], and seismic-fiber-reinforced elastomer isolators (FREI) [7], where sufficient strength and flexibility have to be ensured. Hence, the requirement to improve the mechanical performance in specific directions cannot be accomplished by using non-reinforced elastomers [8,9]. Recent studies have revealed that this knowledge is also considered in so-called smart materials with distinct high (or hyper-) elasticity [10,11]. Such flexible composites are a completely new material class, which evolved due to the increasing demand for improved functionality inspired by certain biomimetic approaches [12], as well as the stronger interest in sustainability and decarbonization [13]. The primary advantage of these flexible composites is the ability to tailor physical properties such as deformation, stiffness, and non-linearity over a much wider range than conventional fiber-reinforced rubbers [10,14]. These concepts can be found in some recent developments, such as artificial muscles [15], exoskeletons, or artificial fingers [16], as well as aeroelastic wings [17,18] with significantly large deformations. Generally, it is evident that these applications of fiber-reinforced elastomers are mostly subjected to cyclic loading. According to the current scientific research activities, industrial fiber-reinforced rubber products or other composite material clusters in the field of civil engineering, e.g., FREI or cement-based composite materials, are investigated regarding fracture mechanics aspects [19,20]. Therefore, important composite material properties in terms of toughness or fracture-mechanics-induced damage are crucial for fatigue lifetime assessments [21,22]. In contrast, recent studies confirm that the applications for smart materials with distinct high elasticity mainly involve exposure to semicyclic loading conditions within a quasistatic range, combined with significantly lower numbers of cycles [12,16]. Therefore, the viscoelastic focus is particularly important in this field. Studies focusing on numerical simulation approaches have addressed this problem, especially for hyperelastic elastomers in combination with stiff reinforcing fibers and their interactions (e.g., fiber–fiber interactions and fiber–matrix interactions), which cause several challenges [23,24]. Therefore, the assessment of dynamic properties and the viscoelastic behavior of those composite materials are essential, as these parameters are decisive when generating specifications for subsequent component designs and lifetime estimations [8]. In order to understand and describe the dynamic and viscoelastic behavior of flexible composites, an extensive characterization can be performed by means of dynamic tests of the temperature, frequency, time, or strain level. Using these characterization methods, dynamic mechanical analysis (DMA) [25,26], and step cycle tests [27,28], the composite structure and performance [8] can be efficiently and quickly investigated.

Many publications have focused on DMA tests, including for fiber-reinforced polymers, which have mainly analyzed short fibers combined with natural rubber or thermoplastic matrices [8,29,30]. The use of short fibers has advantages, such as increased material stiffness and strength, but also has considerable limitations caused by the fiber orientation, which cannot be directed in a specific load-optimized manner. The fibers are rather predominantly aligned in the flow direction during the injection molding process [31,32,33] and the bearable load transfer is limited. It has already been proven in previous studies [34] that reinforced fabrics are more beneficial and economical when enhancing the mechanical properties of composite materials comprising fully unidirectional materials or short fibers. For this reason, some studies have been carried out related to the experimental analysis of composites with continuous fibers or textiles under cyclic loading [2,35,36,37]. Nevertheless, these investigations have focused on natural fibers such as hemp, jute, or cellulose, revealing the disadvantage of moisture absorption [36,38]. The step cycle test represents a promising test method for determining the viscoelasticity and entropic elasticity, which are important parameters in the characterization of the mechanical performance of elastomers and fiber-reinforced composites. Under cyclic loading, rubbers are known to exhibit pronounced viscoelastic behavior [39], including stress softening and hysteresis [28,40]. Significant softening is observed after the first cycles, which can be explained due to the fact that the stress values at reloading are significantly lower than the stress obtained in the first cycle at a similar strain [41]. Consequently, if the deformation is not increased stepwise to higher strain levels, an approximately stationary cycle is achieved after repeated loading, which is characterized by an equilibrium state with preconditioned behavior (stationary hysteresis). Some research has been done in this field, focusing on fiber-reinforced elastomers [33,42,43]. Thus, this approach reveals promising potential for the investigation of the viscoelastic behavior of flexible composites, which was already studied extensively by Peel [10], who provided the basis for the fabrication and mechanics of fiber-reinforced elastomers designed for smart material usage. Moreover, flexible composites have large differences in the stiffness and flexibility between the fibers and elastomeric matrix, causing a textile-like performance [44]. In this context, conventional test setups are not appropriate, and therefore special test devices need to be designed, particularly for these materials [45]. In this context, the fiber–matrix connection is crucial, since the interface is essential for the load transmission [46], and thus for the load coupling (as investigated in previous studies [47]).

The aim of this work is to investigate the mechanical properties of tailored fiber-reinforced elastomers under cyclic loading. The focus includes the dynamic mechanical and viscoelastic behavior, combined with the influence on relaxation, in order to provide information about the near-application performance. Since the presence of fibers makes the characterization of materials even more complex, the influence of the fiber orientation was analyzed, together with parameters such as displacement rate, frequency, and temperature. Regarding the step cycle test, a novel clamping device [48] was implemented for flexible composites, providing sufficient clamping at high deformation and guaranteeing that no material damage was caused by the grips and that no slippage occured. In order to investigate the effects of the large difference in mechanical properties between stiff fibers and flexible matrices, glass fibers (GF) combined with polydimethylsiloxane (PDMS) were chosen as the materials for the flexible composites. The knowledge obtained in these tests provides a better understanding of the performance and application of dynamically loaded flexible composites by considering fiber–matrix load coupling effects. This study aims to obtain customized material parameters for subsequent implementation in numerical simulation models, thus enabling the generation of tailored simulations for elastomeric composites with pronounced textile behavior and high flexibility, followed by the establishment of realistic prediction models of viscoelastic behavior [24,49]. Based on the findings of this study, further applications can be realized by considering other composite material clusters for subsequent fatigue and fracture mechanics life assessments, which can be dealt with using Weibull models [50].

## 2. Materials and Methods

### 2.1. Materials

A commercial E-type GF-fabric supplied by CS Interglas AG (Erbach, Germany) was chosen as reinforcement from a single batch, with a 2/2 twill weave and an area weight of 220 g/m^2^ ± 5%. The standardized yarn classification of the GF was EC9-68xt0, with an indicated twine thickness of 68 tex (grams per kilometer), a mean fiber diameter of approximately 10 µm, and an area bundle distribution of 50/50 in the 0°/90° direction, respectively. The PDMS matrix material, which is available as a cast elastomer (Elastosil RT601 A/B), was chosen for the flexible composite laminates and was provided by Wacker Chemie AG (Munich, Germany). This elastomer is a two-component system (the prepolymer as part A and the crosslinking system as part B) with a density of 1.02 g/cm^3^, a viscosity (in uncured mixed state) of 3500 mPas (at room temperature), and a pot life of about 90 min at room temperature, which was prepared according to the supplier’s specifications at a mixing ratio of 9:1 (part A/part B). As recommended by the manufacturer, the matrix material was then cured in an air circulating drying oven at 70 °C for 60 min. Regarding the mechanical properties of the individual components (i.e., fibers and matrix) and the flexible composite, corresponding tensile tests were carried out as in previous studies [51]. The mechanical properties of the fibers were tested in tensile tests according to ASTM D2256 [52], the elastomeric matrix with ISO 37 [53], while the composite material was investigated based on ISO 527-4 [54]. Thus, an elongation at break value of about 108.6% was determined for the silicone, with a corresponding stress at break value of about 4.5 MPa. Compared to this, the pure fiber material showed an elongation at break value of approximately 2.3%, with a force at break value of about 119.8 N. Subsequently, the mechanical properties of the tailor-made fiber-reinforced elastomers were analyzed regarding the influence of the fiber orientation and adhesion properties. Representative for the flexible composite, the ±45° orientation revealed a determined elongation at break value of about 38.2%, with a corresponding stress at break value of approximately 34.3 MPa [51].

### 2.2. Preparation of Specimens

**Figure 1:**
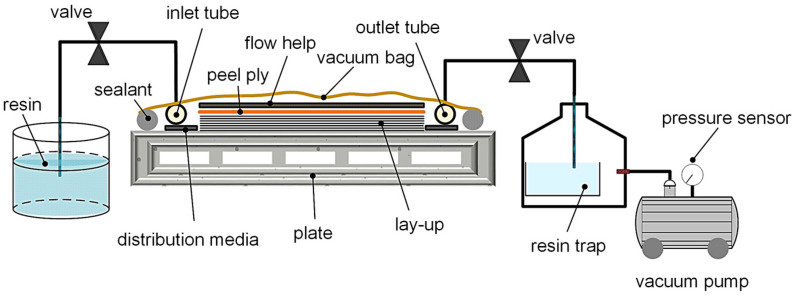
Schematic build-up of the applied vacuum resin infusion (VARI) process for the production of flexible composite plates [1].

For the manufacturing step, the commercial vacuum resin infusion (VARI) process [1] was chosen to prepare the flexible composite specimens, which is schematically illustrated in Figure 1. The VARI process offers advantages in terms of economical production and high individuality, especially at the laboratory scale or for the production of prototypes. Basically, the infusion process comprised two mold halves: a rigid mold on the lower side and a flexible upper mold half, which is the vacuum bag itself. In this investigation, a glass plate was chosen as the rigid lower mold due to its smooth surface and chemically advantageous properties during the infusion. The applied mold release agent (Mono-Coat 1625W) was provided by Chem-Trend GmbH (Maisach, Germany). In order to achieve a linear flow front and considering the size of the manufacturing process, the inlet tube (related to the resin side) and the outlet tube (connected with the vacuum part) were placed at opposite each other on the glass plate. Furthermore, this arrangement enabled optimized inter- and intralaminar impregnation, as well as adequate consolidation quality due to the limited maximum feasible pressure difference of about 10^5^ Pa (atmospheric pressure) [1,55]. For the placing order of the layer structure and the fiber orientation of the woven fabric, the dry textile was cut with a professional cutter G3 M-1600 by Zuend Systemtechnik AG (Altstaetten, Switzerland). This cutter is equipped with a vacuum table to avoid drape defects or unwanted fiber undulations during the cutting procedure. Among the applied disposable materials, such as the flow help, distribution foil, perforated release film, and peel ply, the last layer of the total assembly was constituted by the vacuum bag (outer encapsulation). Moreover, permeable lines were added under the vacuum bag next to both tubes (inlet and outlet) to provide relatively fast media distribution at the beginning of the infiltration, while maintaining a linear progression of the flow front. In this context, a complete impregnation with the prepolymer-based mixture matrix cannot be obtained without the presence of a vacuum. Additionally, further advantages were achieved by using a vacuum, such as (i) the active compacting pressure resulting from the pressure gradient between the vacuum and atmosphere, (ii) uniform layer thickness, and (iii) good consolidation. These aspects are essential to ensure optimized laminate quality.

In terms of the infusion procedure, the prepolymer and crosslinking systems were first mixed and subjected to a degassing step prior to the infiltration. The higher viscosity of the prepolymer compared to classical thermosets at processing temperature in an uncured state raises serious problems, such as causing more complex and challenging impregnation (causing a higher flow resistance and a lower flow rate) because of the pressure gradient (described in D’Arcy’s law for one-dimensional flow form [1]). For this reason, the pot life or infusion time window is negatively affected, which implies the need for a suitable flow to help minimize the risk of incomplete cavity filling or potential porosity. After the infusion, the inlet and outlet vents were clamped to maintain a stable vacuum during the curing step, which was carried out under the same conditions suggested for the curing of the pure elastomeric matrix (60 min at 70 °C in an air circulating drying oven). After the careful demolding of the crosslinked PDMS reinforced with GF (GF-PDMS) composite plates, strip-shaped specimens were produced with the cutter. DMA tests were carried out in tensile loading conditions with the corresponding sample preparation according to ISO 6721-1 [56]. The step cycle tests were also performed under tensile loading, using rectangular samples with a defined length/width ratio of 90:30 (l is the specimen gauge length, between the clamping fixture), ensuring smooth data recording without any effects caused by the clamping area due to affected deformation or hindered fiber reorientation. This sample geometry was already applied in a previous study [51], where the influence of the sample geometry on structural properties, the effects of fiber orientation on shear stresses, and the tensile properties of flexible composites were investigated. For all tests, a defined fiber volume content of about 50% was set, which was verified through thermogravimetric analysis (TGA). Furthermore, to exclude batch variations, only measurements of the same batch were chosen for the DMA and step cycle tests.

### 2.3. Dynamic Mechanical Analysis

**Figure 2:**
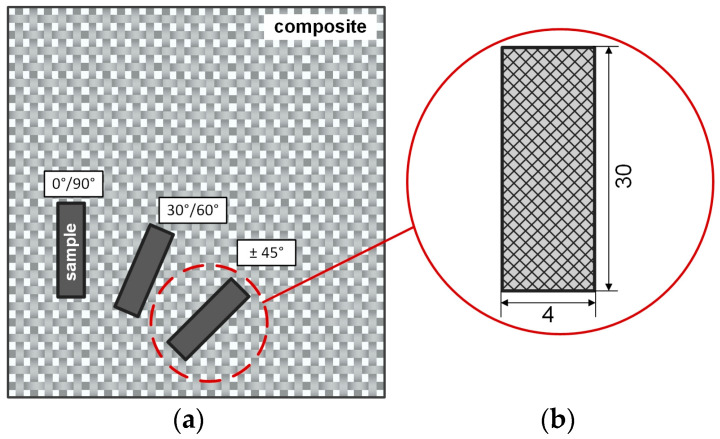
Schematic illustration of the specimen preparations with different fiber orientations (**a**) and the specimen used in the dynamic mechanical analysis in tension mode (**b**).

In this study, dynamic mechanical tests were performed to determine the viscoelastic behavior of fiber-reinforced elastomers. Therefore, the influence of different fiber orientations on the stiffness and entropic elasticity, as well as the impact of frequency variation on the mechanical properties, were investigated in detail. The effects of different fiber orientations of the reinforcement structure and changes of fiber angles related to the load direction were analyzed in previous studies using a methodically validated test plan [44]. In this context, composite samples with a defined warp and weft yarn configuration of ±45°, 30°/60°, and 0°/90° (versus the loading direction) were considered. Figure 2a schematically illustrates how they were obtained from the composite plate. The samples considered in this work were set with a length l of 30 mm, a width w of 4 mm, and a thickness of about 0.35 mm, (see Figure 2b), according to S2 tensile specimens in the ISO 4664 standard [57]. The tests were performed on a Perkin Elmer DMA 8000 (Perkin Elmer VertriebsgmbH, Brunn am Gebirge, Austria). The evaluation and calculation steps were carried out with the corresponding software package Pyris Instrument Managing Software (Perkin Elmer VertriebsgmbH, Brunn am Gebirge, Austria). The storage and loss moduli, as well as the loss factor, were calculated according to ISO 4664. All measurements were carried out in tension mode using a frequency of 1 Hz at a clamping distance of 10 mm. The tests were carried out in temperature ramp mode in the range of −80 °C to +100 °C, with a heating rate of 3 K/min. A static force of 0.3 N was applied and a displacement oscillation amplitude of 5 µm was set. At least five specimens were used for each setting to ensure sufficient repeatability. As reference values for the following data interpretation, the mean value of each setting was determined, along with the corresponding standard deviation.

### 2.4. Step Cycle Test

**Figure 3:**
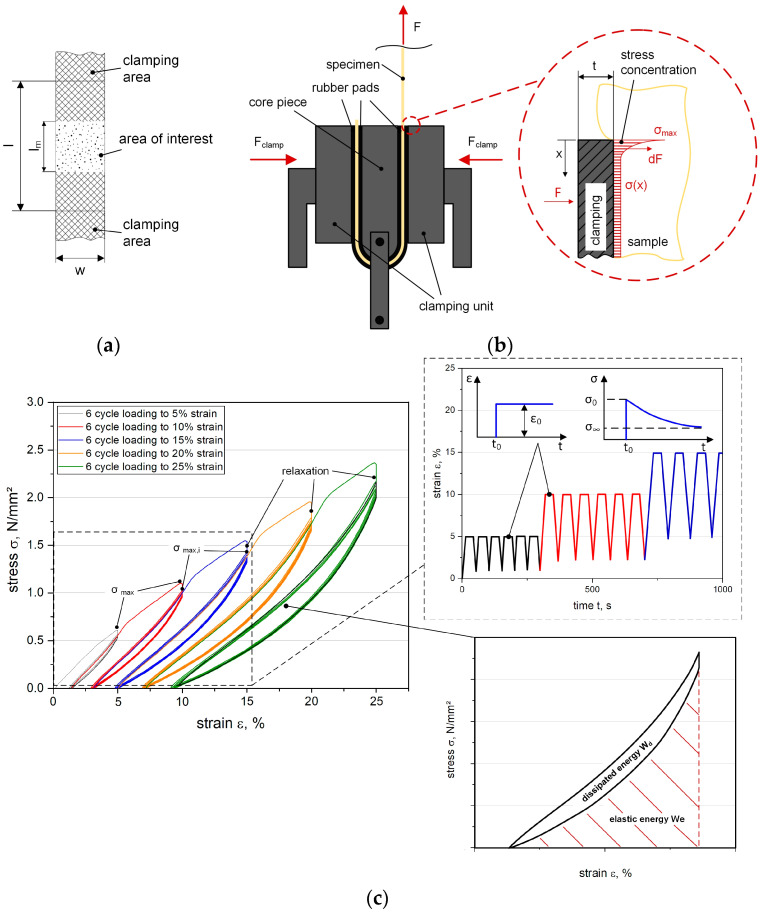
Specimen used in step cycle test (**a**) with the novel clamping device (**b**) [48], schematic build-up of the step cycle test ((**c**), left), and the relaxation and hysteresis diagram ((**c**), right) [59].

The tests were carried out according ISO 527-4 [54] at standard atmosphere conditions according to ISO 291 (20 °C, 50% r. h.) [58] with a universal testing machine Z010 (Zwick Roell GmbH and Co. KG, Ulm, Germany) equipped with a 10 kN load cell. A gauge length l of 90 mm was selected, along with a defined displacement rate v (10 mm/min, 100 mm/min, and 1000 mm/min) to investigate the influence of the strain rate dependency within a wide range according to ISO 37 standard for elastomers, as well as ISO 527-4 standard for composites. The measurement length l m was set to 30 mm. Due to the high flexibility of the composites, the measurement lengths were recorded with an optical image correlation system Prosilica GT 6600 (Allied Vision Technologies GmbH, Stadtroda, Germany) and a fine pattern was sprayed on the sample surface, as depicted in Figure 3a.

The experimental characterization of fiber-reinforced composites using soft matrix materials revealed several problems, since the load transfer into the fibers and the surrounding matrix had to be ensured simultaneously to ensure the complete cross-section area of the sample underwent a homogeneous deformation. Because the substitution of classical thermosets by elastomeric matrix materials in fiber-reinforced polymers leads to highly flexible and almost textile-like material behavior, conventional grippers are not suitable. Thus, a novel clamping system was developed to avoid slippage or failure caused by the grippers, while maintaining sufficient adhesion. This can cause new challenges, such as clearly visible necking of the specimen, particularly in the transition area (clamping gauge region), which increases the risk of slippage and failure of the clamps. Hence, the clamping force F_clamp_ has to be increased, which induces local stress *σ*_max_ caused by the required minimum clamping force. Subsequently, this can lead to fiber damage or preliminary failure. The novel patented device [48] consists of a combination of flat surface clamping using a compression force with an implemented deflection, which is schematically illustrated in Figure 3b. Moreover, due to the sensitivity of the flexible composite, additional rubber pads need to be placed in the clamps. These pads enable prevent slippage and also reduce local compressive stresses, especially in the edges (near-clamping region) of the sample. This is mainly related to the multiaxial stresses caused by large necking and deformation due to the sharp transition in the material between clamping and testing areas. The effects of these local stress concentrations, including their prevention by rubber pads, were already analyzed in previous research studies [51].

Deformation-induced stress softening is an important phenomenon that can be observed during the deformation of reinforced elastomers when tested in cyclic loading. Typically, stress softening can be determined by stretching the elastomer to a certain strain level, followed by unloading and reloading to the same strain level for a second time—the force required to deform the elastomer in the second loading step is lower than that in the first one. Due to this, the specimens were periodically stretched up to a certain strain level and the stress–strain behavior during the loading and unloading steps was recorded. The dissipated energy W_d_ (hysteretic area between the loading and unloding curves of each cycle) can be calculated as the difference between the total absorbed energy W_t_ (integration of the stress–strain response during loading phase) and the stored (elastic) energy W_e_ (integration of the stress–strain response during unloading phase) [60], which is given in Equation (1) and graphically descripted in Figure 3c:

(1) $$W_{d}=W_{t}-W_{e}$$

In this study, the basic concept of the test procedure is to combine the common step cycle method, which was established for quasistatic loading–unloading tests, with an additional relaxation sequence between the loading and unloading phases before the next loading cycle is initiated. This offers the possibility of determining the stress softening between the cycles and the relaxation decrease gradient per cycle, so that a correlation between the current maximum stress value and the corresponding level of relaxation can be observed. Each specimen was stretched and displacement-controlled up to five fixed strain values ε_n_, ranging from 5% up to 25%: at each strain level, each specimen was looped six times. Based on the knowledge gained from previous tensile tests (see Section 2.1), the lower and upper limits were defined to ensure the viscoelastic material behavior was exclusively within the test range [51]. An intermediate holding step at each ε_n_ of 0 s, 10 s, and 30 s was implemented after each loading phase. The unloading step was carried out down to 0.1 N to avoid slackening. A graphic illustration of the measurement procedure is shown in Figure 3c using an exemplary hysteresis loop, where the elastic deformation and the additional relaxation phase are conducted and displacement-controlled. To calculate the stress softening, the decrease of the stress level Δ*σ* (see Equation (2)) was determined as the difference between the maximum stress *σ*_max *n*,*i*_ (first cycle) and the following maximum stresses *σ*_max *n*,*i*+1_ (subsequent cycle number *i*) for a defined strain value ε_n_. Furthermore, the intermediate relaxation sequence *f*_*σ*,*relax*_ was calculated as the stress decrease (vertical load drop) at a constant strain during the holding step of each cycle for a defined strain value ε_n_, which was evaluated with Equation (3):

(2) $$\Delta \sigma =\sigma_{\max n,i}-\sigma_{\max n,i+1}$$

(3) $$f_{\sigma ,relax}=100\frac{\sigma_{\max n,i}-\sigma_{relax\;n,i}}{\sigma_{\max n,i}}$$

For comparability and data reduction, the step cycle tests focused on fiber orientations of ±45° and 30°/60° to assess the impact on the shearing behavior. The main reason for choosing these orientations is reflected by the strong influence of shearing with different fiber orientations when the loading direction differs, especially during deformation of woven textiles. Constituted specimens with a definite length-to-width radio of 3:1 were chosen to guarantee a stress-free area of interest (see Figure 3a). This allowed the load coupling mechanism and the shearing behavior from the fiber–matrix adhesion to be investigated. Moreover, studies [45] revealed that the maximum in-plane deformation is limited by the fiber orientation until the displacement of the fibers relative to each other reaches the maximum shift angle (also known as “locking angle”), where wrinkling perpendicular to the textile plane (the so-called “trellis effect”) emerges, leading to premature fiber breakage [44].

## 3. Results and Discussion

### 3.1. Dynamic Mechanical Analysis

**Figure 4:**
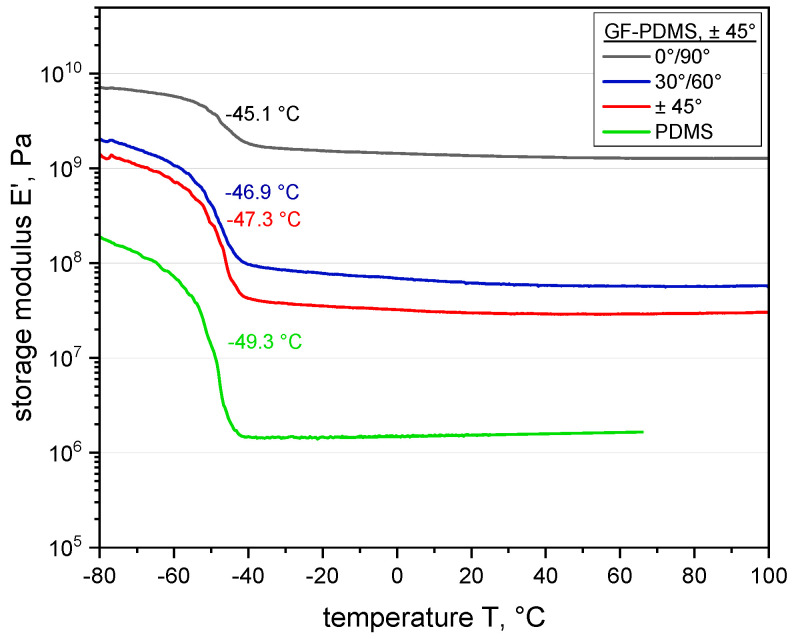
Comparison of storage moduli of glass fiber–polydimethylsiloxane (GF-PDMS) composites with different fiber orientations and polydimethylsiloxane obtained from dynamic mechanical analysis.

Focusing on the study of the stiffness and damping behavior in the entropic elastic region, the influence of different fiber orientations, as well as the effect of the composite interface, the storage modulus was characterized as a function of the temperature, whereby the storage modulus E′ represents the elastic component of the material behavior and is, thus, associated with the material stiffness. Furthermore, the behavior of a fiber-reinforced elastomer (GF-PDMS) was compared with a non-reinforced elastomer (PDMS) to assess the reinforcing effect of the fibers. In Figure 4, the temperature dependence of the composite storage modulus E′ is compared with different fiber orientations in the application range between −80 °C and +100 °C. As is known from literature, the glass transition temperature (Tg) of PDMS is approximately −110 °C [59]. The transition step at about −50 °C is related to the melting of crystalline sequences, which are formed upon cooling due to the highly linear polymer structures [61].

Overall, the results show that the storage modulus increases significantly when reinforcing PDMS with GF and that the reinforcing effect is more significant in the entropic elastic region, as expected. Further, it can be stated that the mechanical properties can be optimized properly, tailoring the fibers reinforcing effect. Nevertheless, the flexibility and characteristic soft regions dominated by the elastic matrix are retained [62]. Furthermore, the results reveal a significant dependence of the composite performance on the fiber orientation. As expected, the fiber-dominated 0°/90° orientation leads to the highest storage modulus compared with the other two orientations, however a small decrease in the modulus can be seen in entropic elastic region. This could be explained by the fact that even with the fibers fully aligned in the loading direction, the fiber–matrix interface and the surrounding matrix have a considerable impact on the load coupling mechanism. The difference in the storage modulus between the 30°/60° and ±45° composites is related to the lay-up of the fiber orientation (asymmetric versus symmetric), causing a significant impact on the load transfer in the fabric. This could be explained by the fact that both composites are more strongly dominated (fiber orientation versus loading direction) by the matrix than the composite with the 0°/90° orientation, which results in the storage modulus being on a lower level. Accordingly, the load transfer between the weft and warp yarns in the fabric is primarily induced via shearing. Moreover, the influence of the fiber–matrix adhesion at the interface is affected by the elastomeric matrix. A detailed discussion of the shear induced load coupling mechanism by different fiber orientations can be found in [45].

### 3.2. Step Cycle Test

**Figure 5:**
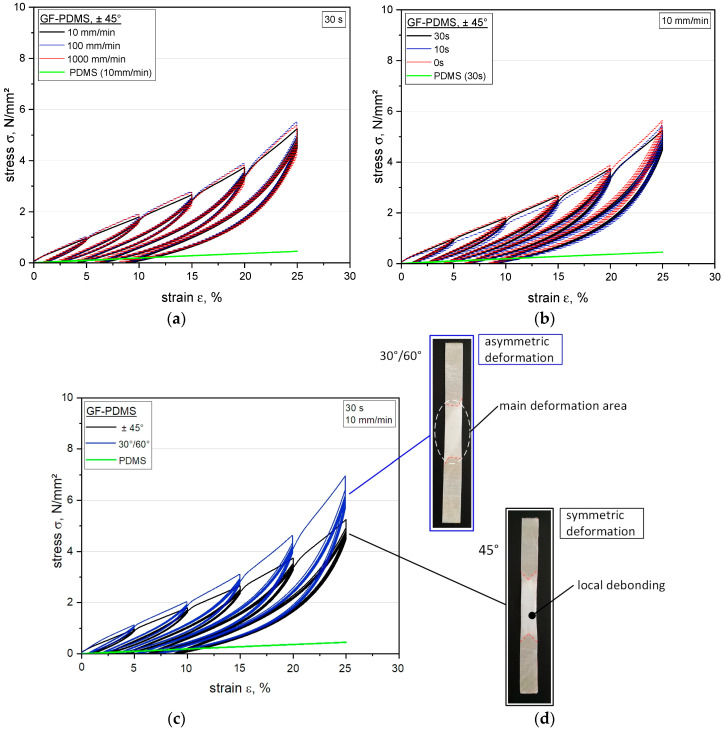
Comparison of stress–strain curves obtained from step cycle tests of glass fiber–silicone composites and silicone at different displacement rates (**a**), relaxation times (**b**), and the fiber orientations (**c**) with corresponding light microscope pictures after the tests (**d**).

Since the viscoelastic properties of dynamically loaded elastomers have a decisive influence on their entropic elasticity, the hysteresis delivers information about the load coupling mechanism [62]. Apart from showing the reinforcing effect of fibers on the elastomeric matrix, the viscoelastic analysis conducted by means of step cycle testing could also indicate the dependence of the damage behavior on the stress level, deformation rate, and relaxation time. Related to this, the step cycle tests conducted on fiber-reinforced elastomers show the typical shape of a stress–strain curve with indicated stress softening, which is illustrated in Figure 5, where data relevant to the tests (performed at different displacement rates) are reported for comparison.

Generally speaking, it can be observed that: (i) no significant dependence of the maximum stress on the applied strain level can be seen in the first loading cycle; (ii) significant stress softening is observed with increasing cycle numbers, especially after the first cycle; (iii) since the unloading path is not affecting by cycling, the first cycle shows the largest hysteresis, and thus the highest amount of dissipated energy. For the study of the strain rate effect (see Figure 5a), a relaxation time of 30 s was adopted in the stress relaxation step, since conventional relaxation tests that had been previously conducted on flexible composites revealed an almost total stress release of more than 95% in this time frame. The slight differences observed between the stress–strain curves measured at different displacement rates could be related to experimental deviations caused by statistical influences (see Figure 5a). The non-reinforced elastomer tested at 10 mm/min showed no hysteretic behavior, which reflects its entropic elasticity, and thus its high rebound resilience [61,62]. This could be explained due to the hyperelasticity of unfilled silicone elastomers [10]. In contrast, the GF-PDMS composites have a distinctly retarded strain recovery ability that was observable under cyclic loading, indicating the occurrence of some dissipative phenomena (e.g., reduction in mechanical properties) during material deformation [63]. This can be explained by several factors, such as (i) the dissipation of energy in the fabric due to fiber–fiber friction, (ii) a weakened fiber–matrix interface due to local adhesion defects, or (iii) deformation and reorientation of fibers (strongly affected by induced shearing during loading, when the extent of fiber angle changes and in-plane deformation increases). In Figure 5d, the weaker fiber–matrix interaction caused by the emergence of several slight local detachments from the surrounding matrix in the interface area is indicated due to the different refractions of the light, whereby the corresponding fiber orientation can be predicted. This favors the formation of wrinkling as a typical behavior of textile-like composites with high flexibility, which further affects the local debonding between the fiber–matrix interaction and tends to augment viscoelastic behavior [23,64]. The results regarding the fiber–matrix interaction, pull-out behavior, and microscopy pictures of the impregnation quality of a fiber bundle with the surrounding matrix were investigated, while the effects of different fiber orientations induced by shearing and their consequences on the load coupling mechanism in flexible composites were analyzed in previous investigations [45]. With respect to the influence of the relaxation time, the experiments with 30 s relaxation time demonstrated slightly lower maximum stress values (at 25% strain) compared to those with 0 s or 10 s relaxation times, as depicted in Figure 5b. This response could be related to the viscoelastic behavior of the elastomeric composite or to damaged induced by the local stress concentration of the fiber–matrix interface, so that recognizable stress softening is only visible above a certain relaxation time (see Figure 5b). Generally, it is evident that no significant difference related to the applied strain rate or relaxation time can be seen in the maximum stress values or for the significant stress softening in the first loading phase, which is clearly observable between the first and second cycles (see Figure 5). Accordingly, the hysteresis area W_d_ and corresponding dissipated energy reveal are highest in the first cycle for all strain levels. This beneficial finding can be adapted to influence either the displacement rate or the different relaxation sequences.

Regarding the influence of the fiber orientation on shear stresses, as displayed in Figure 5c, the results clearly show that a decreasing fiber angle from ±45° to 30°/60° leads to an increased stiffness, and thus to a higher bearable load at equal strain levels. A comparison between both fiber orientations demonstrates that the maximum stress up to a strain of 10% differs only by about 0.3 N/mm^2^ (approximately 17.5%), whereas at a higher strain level of 25%, a significantly increased stress level can be reached with the 30°/60° orientation of about 7.1 N/mm^2^, leading to a 1.9 N/mm^2^ (approximately 36.5%) higher stress value than that obtained with the ±45° orientation. These findings show that despite an improved stiffness, the flexibility (given by the matrix) is maintained at a fiber orientation of 30°/60°, which has a positive effect on the fiber–matrix interface and further on the load-coupling mechanism. In this context, the material performance from step cycle tests reflects the behavior investigated with the performed DMA. In contrast, the ±45° orientation shows more matrix-dominated properties, which can be explained by the large difference between the fiber orientation and the loading direction. Thus, a larger locking angle and increased in-plane deformation inside the composite can be achieved, however this leads to more limitations regarding the maximum bearable stress level.

As an overview, the results of the step cycle tests on GF-PDMS composites are reported in Table 1, investigating decreases in the stress levels Δ*σ* and intermediate relaxation sequences *f*_*σ*,*relax*_ for different values of the applied displacement rate, selected cycle loops, and maximum strain levels. The results show that a direct correlation between the stress level decrease Δ*σ* and the corresponding strain level ε is given, so that with a higher strain the Δ*σ* also increases. Moreover, the stress difference between the first and the second cycles reveals a higher stress decrease Δ*σ*, showing more significant stress softening than between the fifth and sixth cycles until a new equilibrium (stable) state with a repeatable hysteresis loop is achieved. These findings can additionally be related to the relaxation sequence *f*_*σ*,*relax*_ following the same trend.

**Figure 6:**
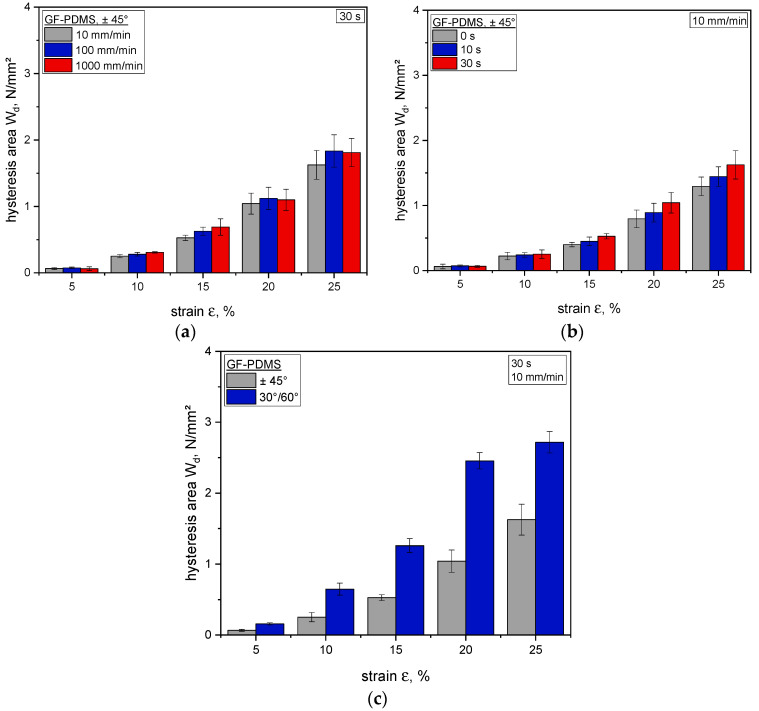
Comparison of hysteresis area (W_d_) obtained from the sixth cycle (new equilibrium state) of the step cycle tests of glass fiber–silicone composites, dependent on the displacement rate (**a**), relaxation time (**b**), and fiber orientation (**c**).

The dissipated specific energy values W_d_ measured for GF-PDMS composites for the sixth cycle of step cycle tests (new equilibrium state) are plotted in Figure 6 as a function of the strain. As depicted, the amounts of dissipated energy (hysteresis area W_d_) and viscoelastic behavior change with higher strain levels.

As expected on the basis of the above reported results, different displacement rates considered at equal strain levels ε have a minor impact on the resulting hysteresis area, and thus on the dissipated energy. Based on this, a mean value of dissipated energy can be inferred, while the viscoelasticity and rebound resilience exhibit no appreciable strain rate dependency. Regarding the influence of different relaxation times on the viscoelastic performance (see Figure 5b and Figure 6b), tests performed at 10 mm/min were considered. As seen before, the hysteresis area W_d_ increases with higher strain levels ε. Furthermore, it is observable that the duration of the stress relaxation step performed at the end of each loading ramp has a clear influence on the material dissipative behavior when it is then cyclically strained up to the same strain level as for the stress relaxation test. For example, at 25% strain, comparing between data relevant to relaxation times of 0 s and 30 s shows that the hysteresis area differs by more than 45 N/mm^2^. Moreover, it is evident that the relaxation time has a significant effect on the hysteresis area—by increasing the relaxation time, the dissipated energy becomes larger, which leads to a decreasing rebound resilience. Further, this finding implies a reduced strain recovery, resulting in a pronounced residual strain. A reason for this behavior could be related to the viscoelastic nature of the elastomer matrix, meaning that the composite has more time to realign under tension loading, which leads to a new state due to the decreased stress, while the residual strain and dissipated energy increase (also reflected by the hysteresis area). Another possible explanation for this could be the energy dissipation due to fiber friction, as well as the presence of a locally affected interface within the fiber–matrix adhesion favored by small defects or by already existing local detachments. Due to the contrary mechanical properties of the individual components, another factor could be the elevated inherent stiffness of the reinforcement structure, which is difficult to overcome or control compared to the hyperelastic matrix. A possible approach could be the use of special surface-treated fibers that offer optimized fiber–matrix adhesion, thus minimizing these effects. However, positive effects on the viscoelastic behavior and permanent irreversible deformation could also be possible.

In Figure 6c, the strong influence of the fiber orientation on the mechanical properties and structure–property interactions is demonstrated. The results reveal that the influence of fibers (e.g., fiber friction or fiber–matrix adhesion) is especially amplified due to the orientation, which exerts a significant effect on the viscoelastic behavior, reflecting the findings obtained from DMA (see Figure 4). In this context, the 30°/60° fiber orientation enhances the stiffness, while the corresponding amount of dissipated energy (at higher strains) also increases (in comparison with the ±45° fiber orientation). The results show that the mechanical and viscoelastic properties of fiber-reinforced elastomers are strongly influenced by the relaxation time and the fiber orientation, which strongly contribute to the final load coupling mechanism in flexible composites.

## 4. Conclusions

The aim of this research was to investigate the mechanical and viscoelastic properties of tailored fiber-reinforced elastomers subjected to cyclic loading. The presence of endless fibers imparts additional complexity in terms of the characterization and interpretation of the properties of flexible composites. Since the research interest in “smart materials” is constantly growing, endless-fiber-reinforced elastomers with high flexibility, in particular with silicone as the matrix material, were studied exclusively in this work. Dynamic mechanical analysis and modified step cycle tests were conducted. To investigate strain-induced stress softening and the stress relaxation behavior of the composites, step cycle tests were implemented. Additionally, a novel testing device was developed to enable the testing of highly flexible elastomer composites. A methodical test plan was elaborated to study the impacts of various relaxation times, displacement rates, strain levels, and different fiber orientations on the composite properties. Furthermore, the impacts of these parameters on the viscoelastic behavior and the effects on the reversible energy and irreversible dissipated energy were assessed.

The results of the dynamic mechanical tests demonstrate that the mechanical properties can be optimized in a specific manner depending on the fiber orientation. In this context, the stiffness can be controlled and improved without significantly impairing the properties of the matrix material (such as flexibility or structure–property interaction for the glass transition and melting temperature range). In general, all step cycle tests showed that although fibers reinforce the elastomer matrix and increases the stiffness, they also contribute to the viscoelastic behavior, which is not evident in the neat matrix, when strained in similar loading conditions. It was found that a higher strain level and relaxation time lead to an increase of the dissipated energy. In contrast, the variation of the displacement rate revealed no impact on the dissipated energy. Tests on flexible composites with different fiber angles revealed an increase of the stiffness (36.5% higher stress at 25% strain) when going from composites with a ±45° to composites with a 30°/60° fiber orientation, while barely reducing the flexibility of the composite. Finally, this reveals adequate correlations between different composite structures and various loading conditions in terms of the cyclic performance. Hence, this study contributes to better understanding the performance of elastomers reinforced with endless fibers, and therefore will help in developing tailored flexible composite materials. It also assesses the structure–property interactions of endless-fiber-reinforced elastomers and emphasizes the importance the effects of tailored load coupling mechanisms of fiber-reinforced elastomer composites on material properties.

Further research is currently ongoing, focusing on the fracture mechanics behavior of flexible composites and investigating significant parameters such as the toughness and dissipated energy due to breakage or fiber–matrix-interaction-related failure, including the effects of tailored surface-treated fibers.

Further studies are in progress to develop an accurate simulation model for cyclic-loaded fiber-reinforced elastomers, considering their viscoelastic behavior using data obtained from this study. This material model will help to simulate the material behavior and failure mechanisms of flexible composites more precisely, enabling optimization and upscaling with regard to component-like applications. Further research is already ongoing to investigate the dependence of the fiber surface on the load coupling mechanism. Therefore, different chemical surface modifications will be applied and their impacts on the cyclic performance and structure–property interactions will be studied.

## Figures and Tables

**Table 1:** Decreases of the stress levels (Δ*σ*) and the intermediate relaxation sequences (*f*_*σ*,*relax*_) for different displacement rates and for selected maximum strain values and cycle numbers in step cycle tests of glass fiber–silicone composites with ±45° orientation.

V, mm/min	ε, %	Δ*σ*, N/mm^2^			*f*_*σ*,*relax*_, % (30 s Relaxation)		
		Cycles 1–2	Cycles 2–3	Cycles 5–6	Cycle 1	Cycle 2	Cycle 6
**10**	5	0.07 ± 0.01	0.03 ± 0.00 *	0.01 ± 0.00 *	6.31 ± 0.15	3.24 ± 0.22	1.51 ± 0.04
	15	0.10 ± 0.02	0.06 ± 0.01	0.03 ± 0.00 *	7.23 ± 0.08	3.43 ± 0.25	2.35 ± 0.04
	25	0.53 ± 0.10	0.31 ± 0.08	0.06 ± 0.01	8.91 ± 0.11	4.11 ± 0.19	2.12 ± 0.03
**100**	5	0.07 ± 0.01	0.05 ± 0.00 *	0.02 ± 0.00 *	9.35 ± 0.60	2.91 ± 0.10	2.23 ± 0.09
	15	0.11 ± 0.05	0.06 ± 0.01	0.01 ± 0.00 *	10.51 ± 0.31	3.85 ± 0.21	3.52 ± 0.10
	25	0.51 ± 0.11	0.18 ± 0.09	0.03 ± 0.00 *	13.42 ± 0.48	5.31 ± 0.33	3.51 ± 0.12
**1000**	5	0.05 ± 0.00 *	0.02 ± 0.00 *	0.01 ± 0.00 *	8.93 ± 0.43	4.31 ± 0.15	2.12 ± 0.06
	15	0.14 ± 0.03	0.09± 0.01	0.01 ± 0.00 *	11.8 ± 0.52	4.20 ± 0.31	2.80 ± 0.13
	25	0.68 ± 0.10	0.27 ± 0.08	0.03 ± 0.00*	16.26 ± 0.71	5.24 ± 0.19	3.31 ± 0.09

* A certain deviation occurs after the third decimal place, therefore the standard deviation is insignificant.
